# Content validation of a questionnaire to assess the knowledge of pediatricians, family, and community physicians on obesity

**DOI:** 10.1590/1984-0462/2023/41/2022063

**Published:** 2023-03-13

**Authors:** Daniel Servigia Domingos, Eduardo Juan Troster, Teresa Cristina Alfinito Vieira

**Affiliations:** a Universidade de São Paulo, São Paulo, SP, Brazil.; b Faculdade Israelita de Ciências da Saúde Albert Einstein, São Paulo, SP, Brazil.; c Hospital Israelita Albert Einstein, São Paulo, SP, Brazil.

**Keywords:** Obesity, Surveys and questionnaires, Validation studies, Pediatric obesity, Obesity management, Obesidade, Inquéritos e questionários, Estudos de validação, Obesidade pediátrica, Manejo da obesidade

## Abstract

**Objective:**

The aim of this study was to validate the content of a questionnaire in order to assess the attitudes and practices in childcare consultations, knowledge on overweight and obesity, their risk factors, and barriers in addressing the issue by pediatricians and family physicians.

**Methods:**

The Delphi technique was used, with the objective of reaching a consensus on a certain subject, through experts’ opinions. The content validity index (CVI) of each item, axis, and questionnaire was calculated. The inter-rater reliability was calculated using an agreement coefficient suitable for the answer distribution such as Gwet's AC2 with ordinal weight.

**Results:**

A total of 63 experts were invited to assess and give their opinion on the questionnaire. In all, 52 accepted the invitation and analyzed the instrument. After two rounds, the questionnaire reached the proper CVI for the study and was considered complete, with its final version having 40 questions, a final index of 95%, and an inter-rate reliability of 0.905.

**Conclusions:**

This instrument, developed to assess attitudes and practices, knowledge, and barriers found in addressing the obesity by primary care physicians, obtained a CVI greater than 0.8 and an excellent agreement coefficient of the 52 judges. Therefore, its content can be considered validated.

## RESUMO

**Objetivo:**

Validar o conteúdo de um questionário para a avaliação das atitudes e práticas nas consultas de puericultura, para o reconhecimento do sobrepeso e da obesidade, seus fatores de risco e barreiras encontradas para abordar o tema por pediatras e médicos da família.

**Métodos:**

Foi utilizada a técnica de Delphi, com o objetivo de alcançar um consenso sobre determinado assunto, por meio da opinião dos especialistas. Foi mensurado o índice de validade de conteúdo por item, por eixo e para o questionário geral. A concordância entre os avaliadores foi calculada utilizando-se coeficiente de concordância adequado à distribuição de respostas, tal como o AC2 de Gwet com ponderação ordinal.

**Resultados:**

Foram convidados 63 juízes para avaliar e opinar sobre o questionário. Cinquenta e dois aceitaram o convite e analisaram o instrumento. Após duas rodadas, o questionário foi finalizado por atingir o índice de validação de conteúdo (IVC) adequado para o presente estudo. O questionário final terminou com 40 questões, e o índice final do questionário atingiu 95%. O índice de concordância geral entre os juízes foi de 0,905.

**Conclusões:**

Este instrumento, construído para avaliar as atitudes e práticas, conhecimento e barreiras encontrados na abordagem do problema da obesidade por médicos da atenção básica, obteve IVC maior que 0,8 e excelente índice de concordância dos 52 juízes. Assim, seu conteúdo pode ser considerado validado.

## INTRODUCTION

Obesity has become a global public health problem and was classified as the epidemic of the 21st century. In approximately 40 years, the global prevalence of overweight and obesity has increased eightfold in the 5–19 age group.^1^ This has worsened in the past 20 years, with the global prevalence of children with excessive weight having jumped from 4.9 to 5.6% in 2016, reaching 340 million children aged between 5 and 19 years and 38 million children aged under 5 years in 2019, according to the World Health Organization (WHO).^2^

In Brazil, the 2019 National Health Survey showed that the percentage of obese adults increased from 12.2 to 26.8% (from 2003 to 2019), while 26.1% of adolescents aged 15–19 years were overweight.^3^

In the United States, obesity is associated with an annual cost increase of 36% with health and 77% with medication, compared to the expenses of people with normal weight.^4^ In Brazil, in 2018, 16% of the total hospital admissions through the Unified Health System were due to comorbidities associated with obesity, such as arterial hypertension and diabetes, at a cost of BRL 3.84 billion.^5^

According to a 2016 WHO survey, obesity is the fifth leading cause of death, accounting for 23% of ischemic diseases and 44% of diabetes cases globally.^6^ Studies have shown that obese children and adolescents are more likely to remain obese throughout their lives.^7^

Certain periods of childhood are considered critical for the development of obesity, such as the neonatal period, the first year of life, the ages between 3 and 7 years, and adolescence.^8^ Evidence for the period known as “1000 days,” which covers from the day of conception to the second year of life, is increasing. This phase is a window of opportunity for environmental and nutritional interventions in children at risk of obesity, which, if successful, could result in improving their body composition in the long term.

Family physicians and pediatricians are primary care professionals who, by monitoring children and their families regularly from prenatal care to adulthood, can identify overweight and obesity, thus being able to carry out an early intervention that may result in positive changes in the child's weight gain.^9^ The question is whether these professionals find it difficult to detect overweight and obesity and their risk factors at an early stage. An American study applied a questionnaire to primary care physicians and found out that only 26% of them diagnosed obesity correctly, 56% thought they had received sufficient training to prevent and treat obesity, and 63% believe that they have limited time to talk about nutrition.^10^ In 2006, another American study applied a questionnaire to pediatricians with the objective of evaluating attitudes, practices, and barriers found by pediatricians related to obesity. Once identified, a series of policies were proposed to address it. In 2017, the same questionnaire was applied, and an improvement was observed in most of the flaws pointed out in 2006.^11^ The hypothesis raised is whether a questionnaire would be able to measure the knowledge of pediatricians and family physicians regarding the diagnosis of overweight, obesity, and their risk factors, in addition to identifying the professionals’ difficulties in facing the issue. As there is no validated instrument that evaluates the practices, knowledge, and barriers related to the obesity of primary sector physicians, in Portuguese, it was decided to develop an instrument to validate its content and thus identify the aspects that, in primary health care attention, need to be improved in terms of approach, diagnosis, and prevention of overweight, with the objective of contributing to the decrease in obesity prevalence in children and adolescents in Brazil.

## METHOD

This is a study with the subsequent application of questionnaires, aiming to validate an analysis instrument. The Delphi technique was used to judge the information and reach a consensus among experts on the given subject. This technique consists of a series of rounds of questionnaires with controlled feedback, allowing experts (judges) to express their opinions on a given topic, in order to find the most reliable consensus on the topic. It is a useful technique when a group's judgment is more reliable than individual opinions and has the advantage of including geographically distant participants in a simple, quick, and low-cost manner.^12^

To validate the content, some questionnaires were submitted to a group of judges considered experts on the topic in question. The following inclusion criteria were applied: pediatric endocrinologist or pediatric nutrologist, as they are professionals used to deal with obesity. Professionals who do not have qualifications as specialized physicians in the area were excluded from the sample. The sample included professionals from different regions of Brazil with expertise in the area and availability to participate in the study. A convenience sample was used. We met experts known to the authors and asked for indication of other professionals. Those with the highest academic titles and used to treating patients with obesity were prioritized. A minimum number of 20 was initially stipulated to start the study. Due to the fear of dropouts during the project, 63 judges were invited.

An e-mail invitation was sent to each judge with explanations about the research objectives and the Informed Consent Form. The instrument was applied through the online tool *Google Forms*, and the experts had 10 days to reply with their answers.

A Likert scale ranging from 1 (strongly disagree) to 5 (strongly agree) was used to classify the experts’ opinions.^12^ Each question, each topic axis, and the questionnaire as a whole were evaluated as follows.

Each topic was evaluated using four questions created by the authors in order to assess the representativeness, relevance, clarity, and coherence of the content, as these items are the most commonly used criteria in the content validity assessment sheet.^12^

- Is the statement clear? Can it be understood at a first reading?
- Is the answer consistent with the question?
- Are the alternatives clear, without the possibility of double interpretation?
- Is the question relevant, that is, is it important to know the content addressed in the question?

Each axis was evaluated through two close-ended questions and an open-ended one:

- Is this axis relevant? Is it important within the study subject?
- Is this axis comprehensive? Does it cover the main aspects of the topic?
- Open-ended question: Are there any questions that could be included or modified within this axis? Which one?

The questionnaire as a whole was evaluated through two close-ended questions and an open-ended one:

- Is this questionnaire relevant to assess the knowledge of pediatricians and family doctors about obesity diagnosis, risk factors, and the adoption of preventive measures?
- Is it comprehensive? Does the questionnaire cover the main aspects to assess the knowledge about the study topic?
- Open-ended question: Is there anything you would like to add to the questionnaire?

The initial questionnaire had 35 questions distributed in three axes: Axis 1: “Attitudes and practices of pediatricians or family physicians during the childcare”; Axis 2: “Theoretical knowledge about obesity diagnosis and identification of its risk factors”; and Axis 3: “Barriers found by family physicians and pediatricians to address the obesity issue during the appointments.”

Scientific articles, guidelines from the Brazilian Society of Pediatrics, and the WHO were used as references for the preparation of the questions.^13,14,15,16,17,18,19,20,21^

At the end of each round, the content validation index (CVI) was measured. To finish the Delphi technique rounds and validate the questionnaire, the CVI should be greater than 80%.

The CVI measures the percentage of experts who agree on certain aspects of the instrument. The score is calculated by adding up items that were marked with a “4” or “5” by the experts, dividing this number by the total number of responses, and multiplying the result by 100. New Delphi rounds were carried out until reaching 80% CVI.

In the characterization of the experts, the categorical variables were described by absolute and relative frequencies and the numerical variables by means or medians and standard deviations or interquartile range.

In the evaluation phase using the Delphi technique, the experts’ answers were classified by absolute frequencies and percentages, to verify the clarity of alternatives and statements, the coherence between statements and alternatives, and the relevance and pertinence of the questions.

The questionnaire evaluations were described by the percentages of each type of answer. The CVI was calculated per item (CVI-i), per axis (CVI-a), and for the full questionnaire (CVI-q), according to the aspect evaluated. The CVI-a is the average of the CVI-i of each item of the questions referring to the analyzed axis, and the CVI-q is the average of the CVI-i of each item evaluated in all the questions. The analysis was performed using the SPSS software, version 24. Data were collected and stored in Google Drive and exported to Microsoft Excel, where the analyses were performed.

The agreement among the experts was calculated using an inter-rater reliability measurement appropriate to the answer distribution, such as Gwet's AC2 with ordinal weight,^22^ with the coefficients followed by 95% confidence intervals and p-values for the test of hypotheses. The agreement coefficients were compared to the classification presented by Altman,^23^ which considers poor the coefficients lower than 0.2, reasonable between 0.2 and 0.4, moderate between 0.4 and 0.6, good between 0.6 and 0.8, and excellent those above 0.8. For this study, CVI values or agreement coefficients over 0.80 were considered satisfactory. The analyses were performed using the irrCAC, an R package.^24^

The survey project was submitted for analysis by the Survey Ethics Committee and approved by Statement No. 4.636.789, CAAE: 43156021.4.0000.0068, issued on April 8, 2021.

## RESULTS

A total of 63 experts were invited to assess and give their opinion on the questionnaire. In all, 52 accepted the invitation and analyzed the instrument, and 11 accepted the invitation but were unable to respond within the deadline. At the beginning of the questionnaire, the experts were asked the following question: “Do you think obesity is handled well by family physicians or pediatricians?” 65.4% disagreed. The data of the 52 experts are presented in Table 1.

**Table 1.** Characterization of the experts.

Number of specialists	52
Median age	47.4 years
Minimum and maximum age	32–79 years
Female	75%
Regions of Brazil	
South	9.6%
Southeast	53.8%
Central-West	13.5%
Northeast	13.5%
North	9.6%
Area of expertise	
Endocrinology	82.7%
Nutrology	17.3%
Experience in the area of expertise (years)	
<5	7.7%
5–15	28.8%
16–25	38.5%
26–35	15.4%
>35	9.6%
Workplace	
Public service only	11.5%
Private service only	5.8%
Private and public services	82.7%
Title	
Specialist title	21.2%
Master's degree	28.8%
Doctorate degree	42.3%
Postdoctoral degree	7.7%
Percentage of overweight patients seen in the week	
<10	27%
11–20	48%
21–30	19%
>30	6%

In the first round, the experts expressed their opinions for each item of the 35 questions, according to the Likert scale. Questions 8 and 9 did not reach the minimum CVI of 80% for clarity of alternatives, and question 31 did not reach the CVI for clarity of statement. Also, 100% of the experts found the three axes relevant, and 98% found them comprehensive. Regarding the questionnaire, 98% rated it as relevant and comprehensive.

After the statistical analysis and reading experts’ comments, the questionnaire was reformulated. In addition to the questions with CVI<80%, questions 1, 29, and 30 were modified based on the experts’ opinions, even having reached CVI>80%. The Likert scale of axis 1 was changed based on suggestions, and four new questions were added. The changes were submitted for analysis by the experts in a second round.

In the second round, the same 52 experts analyzed and answered the questionnaire. In total, 86% of them agreed to modify the Likert scale of axis 1. All modified questions achieved a CVI greater than 80%, and the four added questions obtained a CVI above 90%.

After two rounds, the questionnaire reached the CVI appropriate for the study, with its final version containing 40 questions. The final CVI of all questions was over 80%. The CVI of the axis and the final questionnaire can be found in Table 2.

**Table 2.** Final content validation index of the axis and the questionnaire.

Axis	CVI
1 (questions 1–15)	94.26
2 (questions 16–31)	95.16
3 (questions 32–40)	95.99
Final questionnaire	95.01

After the two rounds, the expert agreement index was calculated using Gwet's AC2 inter-rater reliability coefficient. In the second round, the agreement indexes for clarity of the statements, clarity of the alternatives, coherence, relevance, and general agreement were excellent. After the final changes, the inter-rater reliability was calculated again with 40 questions, and the data are shown in Table 3.

**Table 3.** Inter-rater reliability assessment after the changes in the questionnaire.

Aspect	Agreement coefficient (95% CI)	p-value
Clarity of the statement	0.896 (0.880; 0.913)	<0.001
Clarity of the alternatives	0.843 (0.807; 0.878)	<0.001
Coherence	0.896 (0.877; 0.916)	<0.001
Relevance	0.970 (0.958; 0.981)	<0.001
General	0.905 (0.893; 0.918)	<0.001

## DISCUSSION

The minimum requirement for the development of a measurement instrument is to have its content validated as relevant and representative.^25^ The validation process is one of the most common challenges in the preparation of this type of instrument. This study faced this challenge and validated the content of a questionnaire that will serve as an instrument to assess the attitudes, the practices, and the knowledge of family physicians and pediatricians in relation to the diagnosis, prevention, and treatment of obesity, as well as to identify the barriers found by these professionals to address this topic.

The validity of a measurement tool is a critical factor in its selection and application in both professional practice and academic research. An instrument is valid when it measures what it was supposed to measure.^26^

The content is validated by a panel of experts who judge the instrument elements and categorize the tool according to the relevance and representativeness of its content.^27^ There is no consensus in the literature on the minimum number of experts. Grant and Davis do not specify an exact number, but often the studies use a group of up to 10 experts. These authors also propose that the decision on the number of experts depends on their level of specialization, their experience, and their knowledge on the subject being evaluated.^28^

This study invited 63 experts. There was also a concern about the quality of the experts. Half of them had a doctorate or a postdoctoral degree, setting up a panel of experts with a high degree of specialization. Additionally, they came from all regions of Brazil, contributing opinions based on their different regional realities.

At the end of the study, more than 80% of the experts responded not only to the first round but also to the second. This high number of experts could have been a problem for the study, as it is known that the higher the number of people evaluating a topic, the harder it is to reach a consensus, which could lead to many evaluation rounds. Nevertheless, this was not the case, and a consensus was reached after just two rounds.

The low abstention observed in this study can be explained by the use of an online form that facilitated the experts’ answers and reminded them about the evaluation each week. Another reason that may justify such adherence is the fact that the prevalence of obesity has been increasing in recent years and that its negative impact on individual and collective health has encouraged the experts to contribute with an instrument that can improve the management of such disease in the health system.^1,10,11^

Upon the receipt of the experts’ answers, the questionnaires were subjected to quantitative and qualitative analyses. There is more than one approach to verify the validity of an instrument content, as well as several statistical methods for data analysis. Some studies classify these methods into two categories: indexes related to content validity and indexes of general agreement.^29^ This study used both methods for the analysis.

Polit and Beck proposed a CVI>0.78 for each item of the questionnaire and a mean CVI>0.9 for the content to be considered validated.^30^ This study required an index >0.8 for both measures. At the end of the study, the CVI of all instrument items was above 0.8. The mean CVI of all axes and the mean CVI of the final questionnaire were both above 0.9, which allowed the content of the instrument to be considered validated and representative.

There are several ways to calculate the agreement coefficient. This study chose to use Gwet's AC2, which is more appropriate when there are concentrations of responses in one direction, that is, when the proportion of favorable and unfavorable responses is not the same.^22^ After all the modifications, the experts agreed that the statements and alternatives were clear and that the questionnaire was coherent and relevant, as all these items had an index greater than 0.8 with statistical significance. The final agreement coefficient was excellent, reaching 0.905 in the general analysis, indicating that, despite the high number of experts, they agreed with each other.

As limitations of the study, we can mention the general scope of the questionnaire, which was 0.78 in the first round. A possible justification for this index lower than 0.8 may be the fact that not all the causes of obesity were addressed in the questionnaire — only the main ones, because obesity is multifactorial, and it was not possible to include all causes.^13^ Conducting a pilot test with the application of the final instrument to the target audience could also bring improvements to the tool. The lack of face-to-face meetings with the experts to clarify doubts regarding the evaluation steps could negatively contribute to the results. Therefore, to minimize this risk, the guidelines of some authors were followed for the instrument validation process, such as sending an invitation letter, the references, the study objective, and a detailed instruction manual on how to proceed with the evaluation. The validation process is more comprehensive than content validation.^26^ Therefore, it is necessary to subject the questionnaire to other psychometric tests, such as reliability or construct validation, for example, before applying it in research.

The development of a questionnaire that can assess whether family physicians and pediatricians know how to diagnose obesity and overweight, whether they know how to identify their risk factors, and what are the barriers they find in addressing the issue can be useful in improving pediatric patient care and adopting effective preventive measures against obesity, as these professionals are inserted in a strategic position in the public health system, which is the primary sector.^9^ These physicians are essential in the detection of obesity, the application of preventive measures, and the assistance in and implementation of public policies to fight the increased prevalence of obesity.

This developed instrument had a CVI>0.8, and an excellent agreement coefficient from the judges, which validate the instrument. Therefore, the instrument can be considered relevant to identify aspects that need to be improved with regard to the approach, diagnosis, and prevention of overweight in primary health care attention.

## References

[1] NCD Risk Factor Collaboration (2017). Worldwide trends in body mass index, underweight, overweight, and obesity from 1975 to 2016: a pooled analysis of 2416 population-based measurement studies in 1289 million children, adolescents, and adults. Lancet.

[2] World Health Organization [homepage on the Internet] (2018). Obesity and overweight.

[3] Instituto Brasileiro de Geografia e Estatística [homepage on the Internet] PNS 2019: Quem mais utiliza o SUS avaliou mais positivamente a qualidade dos serviços de Atenção Primária à Saúde.

[4] Sturm R (2002). The effects of obesity, smoking, and drinking on medical problems and costs.. Health Aff (Millwood).

[5] Nilson EA, Andrade RC, Brito DA, Oliveira ML (2020). Custos atribuíveis a obesidade, hipertensão e diabetes no Sistema Único de Saúde, Brasil, 2018. Rev Panam Salud Publica.

[6] Commission on Ending Childhood Obesity (2016). Report of the commission on ending childhood obesity..

[7] Ward ZJ, Long MW, Resch SC, Giles CM, Cradock AL, Gortmaker SL (2017). N Engl J Med.

[8] Ong KK, Loos RJ (2006). Rapid infancy weight gain and subsequent obesity: systematic reviews and hopeful suggestions.. Acta Paediatr.

[9] (2012). Brazil. Ministério da Saúde. Secretaria de Atenção à Saúde. Departamento de Atenção Básica. Child health: growth and development/Ministério da Saúde. Secretaria de Atenção à Saúde. Departamento de Atenção Básica..

[10] Spivack JG, Swietlik M, Alessandrini E, Faith MS (2010). Primary care providers’ knowledge, practices, and perceived barriers to the treatment and prevention of childhood obesity.. Obesity (Silver Spring).

[11] Belay B, Frintner MP, Liebhart JL, Lindros J, Harrison M, Sisk B (2019). US pediatrician practices and attitudes concerning childhood obesity: 2006 and 2017.. J Pediatr.

[12] Humphrey-Murto S, Varpio L, Gonsalves C, Wood TJ (2017). Using consensus group methods such as Delphi and Nominal Group in medical education research.. Med Teach.

[13] Larqué E, Labayen I, Flodmark CE, Lissau I, Czernin S, Moreno LA (2019). From conception to infancy - early risk factors for childhood obesity.. Nat Rev Endocrinol.

[14] Woo Baidal JA, Locks LM, Cheng ER, Blake-Lamb TL, Perkins ME, Taveras EM (2016). Risk factors for childhood obesity in the first 1,000 days: a systematic review.. Am J Prev Med.

[15] Nelson JM, Vos MB, Walsh SM, O’Brien LA, Welsh JA (2015). Weight management-related assessment and counseling by primary care providers in an area of high childhood obesity prevalence: current practices and areas of opportunity.. Child Obes.

[16] Pietrobelli A, Agosti M, MeNu Group (2017). Nutrition in the first 1000 days: ten practices to minimize obesity emerging from published science.. Int J Environ Res Public Health.

[17] Daniels SR, Hassink SG, Committee on Nutrition (2015). The role of the pediatrician in primary prevention of obesity.. Pediatrics.

[18] Koletzko B, Fishbein M, Lee WS, Moreno L, Mouane N, Mouzaki M (2020). Prevention of childhood obesity: a position paper of the Global Federation of International Societies of Paediatric Gastroenterology, Hepatology and Nutrition (FISPGHAN). J Pediatr Gastroenterol Nutr.

[19] World Health Organization (2019). Guidelines on physical activity, sedentary behaviour and sleep for children under 5 years of age..

[20] (2018). Physical Activity Guidelines for Americans.

[21] Kerzner B, Milano K, MacLean WC, Berall G, Stuart S, Chatoor I (2015). A practical approach to classifying and managing feeding difficulties. Pediatrics.

[22] Gwet KL (2014). Handbook of inter-rater reliability: the definitive guide to measuring the extent of agreement among raters.

[23] Altman DG (1991). Practical statistics for medical research.

[24] R Core Team [homepage on the Internet] R: A language and environment for statistical computing. R Foundation for Statistical Computing.

[25] Streiner DL, Norman GR, Cairney J (2014). Health measurement scales: a practical guide to their development and use.

[26] DeVon HA, Block ME, Moyle-Wright P, Ernst DM, Hayden SJ, Lazzara DJ (2007). A psychometric toolbox for testing validity and reliability. J Nurs Scholarsh.

[27] Lynn MR (1986). Determination and quantification of content validity. Nurs Res.

[28] Grant JS, Davis LL (1997). Selection and use of content experts for instrument development.. Res Nurs Health.

[29] Almanasreh E, Moles R, Chen TF (2019). Evaluation of methods used for estimating content validity.. Res Social Adm Pharm.

[30] Polit DF, Beck CT (2006). The content validity index: are you sure you know what's being reported? Critique and recommendations.. Res Nurs Health.

